# Multi-scale numerical simulations on piezoresistivity of CNT/polymer nanocomposites

**DOI:** 10.1186/1556-276X-7-402

**Published:** 2012-07-17

**Authors:** Bin Hu, Ning Hu, Yuan Li, Kentaro Akagi, Weifeng Yuan, Tomonori Watanabe, Yong Cai

**Affiliations:** 1 Department of Chongqing, University of Science & Technology, Daxuecheng, Chongqing, 401331, People’s Republic of China; 2 Department of Mechanical Engineering, Chiba University, 1-33 Yayoi-cho, Inage-ku, Chiba, 263-8522, Japan; 3 Department of Nanomechanics, Tohoku University, 6-6-01 Aramaki-Aza-Aoba, Aoba-ku, Sendai, 980-8579, Japan; 4 School of Manufacturing Science and Engineering, Southwest University of Science and Technology, 59 Qinglong Road, Mianyang, 621010, P.R. China

**Keywords:** Carbon nanotube, Conductive network, Nanocomposite, Piezoresistivity, Tunneling effect

## Abstract

In this work, we propose a comprehensive multi-scale three-dimensional (3D) resistor network numerical model to predict the piezoresistivity behavior of a nanocomposite material composed of an insulating polymer matrix and conductive carbon nanotubes (CNTs). This material is expected to be used as highly sensitive resistance-type strain sensors due to its high piezoresistivity defined as the resistance change ratio divided by the mechanical strain. In this multi-scale 3D numerical model, three main working mechanisms, which are well known to induce the piezoresistivity of strain sensors fabricated from nanocomposites, are *for the first time* considered systematically. They are (a) the change of the internal conductive network formed by the CNTs, (b) the tunneling effect among neighboring CNTs, and (c) the CNTs’ piezoresistivity. Comparisons between the present numerical results and our previous experimental ones were also performed to validate the present numerical model. The influence of the CNTs’ piezoresistivity on the total piezoresistivity of nanocomposite strain sensors is explored in detail and further compared with that of the other two mechanisms. It is found that the first two working mechanisms (i.e., the change of the internal conductive network and the tunneling effect) play a major role on the piezoresistivity of the nanocomposite strain sensors, whereas the contribution from the CNTs’ piezoresistivity is quite small. The present numerical results can provide valuable information for designing highly sensitive resistance-type strain sensors made from various nanocomposites composed of an insulating polymer matrix and conductive nanofillers.

## Background

Due to their excellent electrical properties, carbon nanotubes (CNTs) of high aspect ratio are predicted to open up a whole range of smart structural applications [1]. In particular, it has been confirmed that the conductance of a CNT could be dramatically changed by introducing a mechanical strain as a consequence of structural change (e.g., chirality change [2]). Due to this piezoresistivity of CNTs, great interest has recently been aroused in building strain sensors not only using CNTs themselves (either single-walled carbon nanotube (SWCNT) or multi-walled carbon nanotube (MWCNT)) [3-5] but also incorporating them into an insulating polymer matrix [6-9], as summarized in a recent review paper [10]. As compared to conventional metal-foil strain gauges, higher sensitivities have been observed in these novel sensors at least at a macro-scale [5-9]. Moreover, a linear piezoresistivity has been identified within very small strain ranges (e.g., 200 με [4] or 1,300 με [5]), whereas nonlinear piezoresistivity has also been reported for a quite large strain range (e.g., 6,000 με [6,7]). In spite of these exciting results, the fundamental understanding of piezoresistivity behavior in a CNT/polymer nanocomposite is still lacking, largely due to less effort being put into theoretical and numerical investigations.

Based on a three-dimensional (3D) statistical resistor network model [11], we have previously considered the change of internal CNT conductive network and tunneling effect in exploring the piezoresistivity behavior of the above CNT/polymer nanocomposite strain sensors [12]. The movement of CNTs in the polymer under a given strain is firstly predicted using a rigid-body fiber reorientation model. Then, by integrating this fiber reorientation model into the 3D resistor network model in an iterative way, the resistance change of the nanocomposites caused by the applied strain is estimated. Moreover, the predicted electrical conductivity and piezoresistivity of CNT/polymer nanocomposites are verified using our previous experimental data [6-8]. Furthermore, the influence of various parameters (i.e., cross-sectional area of tunnel current, height of barrier, alignment of CNTs, electrical conductivity, and nanofiller electrical conductivity) are systematically investigated [12]. However, the effect of the piezoresistivity of CNTs themselves is not mentioned.

In the present work, our previously improved 3D resistor network model [12] is further extended within a multi-scale framework in which, *for the first time*, the following three working mechanisms are incorporated simultaneously:

(a) The change of the internal conductive network formed by the CNTs themselves.

(b) The tunneling effects among neighboring CNTs.

(c) The piezoresistivity of CNTs themselves.

To include the CNTs’ piezoresistivity effect in a multi-scale way, we firstly calculate the strain of an embedded CNT by applying a mechanical tensile strain on a micro-mechanics-based cylindrical representative volume element (RVE), which simulates the CNT deformation for the case of multiple one-directionally aligned CNTs in a polymer matrix. Then, we transform the strain of the CNT into that of an arbitrary-oriented CNT dispersed in macroscopic CNT/polymer nanocomposites. By employing this strain information and the piezoresistivity of CNTs themselves predicted from the first-principle computations at the atomistic scale [13,14], we update the electrical conductance of the CNTs in the internal conductive network of the macroscopic nanocomposite sensors under the applied strain. By using this updated electrical conductance of CNTs and incorporating the internal network change of CNTs and tunneling effect simultaneously, we finally comprehensively evaluate the piezoresistivity of the nanocomposite strain sensors.

## Methods

So far, the resistor network model has been well documented [15] to predict the electrical conductivity of some conductive composites. However, because of its high computational cost, the corresponding numerical studies have been limited [15,16], even for that of conventional composites with short carbon fibers. There has been no work about its extension for predicting the piezoresistivity of CNT/polymer nanocomposites, except for our previous work [12]. To maintain the integrity of the present work, our previously improved 3D resistor network model [12] is briefly described.

### An improved 3D resistor network model without the effect of CNT’s piezoresistivity

**Figure 1:**
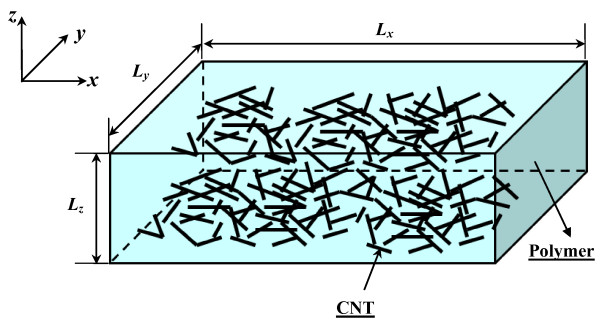
A 3D representative unit cell.

As shown in Figure 1, a 3D resistor network that contains randomly distributed CNTs in a polymer is constructed in a 3D cubic unit cell. Here, the electrical conductive paths in the insulating matrix phase are completely neglected by taking into account the very low electrical conductivity of most polymers. For instance, the electrical conductivity of epoxy resin used in our previous experiments [6-8] is close to 10^−10^ S/m. Moreover, the aggregation of CNTs is also neglected, according to our previous experimental studies [6,7] in which no obvious aggregation is observed in the nanocomposites filled with straight MWCNTs of a comparatively large diameter (≥50 nm).

**Figure 2:**
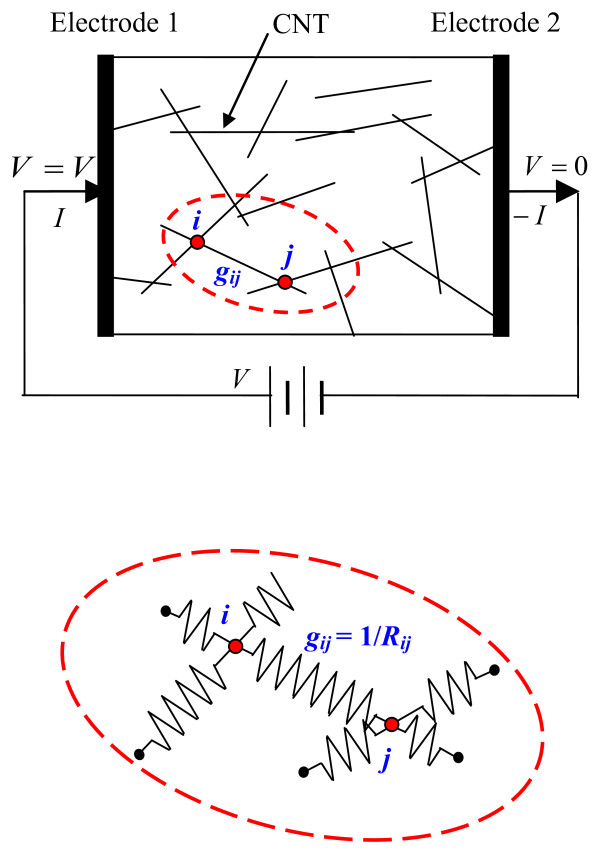
Schematic model of the CNT network.

The CNT was modeled as a cylinder with a length of *L* and a diameter of *D*. For the ideal state of uniformly dispersed straight CNTs in Figure 2, the simulations were carried out using a procedure to identify those CNTs being in contact, until the CNT loading reaches the required volume fraction of the CNTs in the 3D unit cell to form a global conductive network [11]. To construct the 3D resistor network model (a simple 2D model is given in Figure 2), for a CNT with two points, *i* and *j*, contacting with two other neighboring CNTs, the conductance *g*_ij_ between *i* and *j* (the inverse of resistance *R*_*ij*_) can be evaluated as:

(1) $$g_{\mathrm{ij}}=\sigma_{\mathrm{CNT}}\frac{S_{\mathrm{CNT}}}{l_{\mathrm{ij}}}$$

where *l*_ij_ is the length between the points *i* and *j*, and *σ*_CNT_ and *S*_CNT_ are the electrical conductivity and the cross-sectional area of the CNTs, respectively.

**Figure 3:**
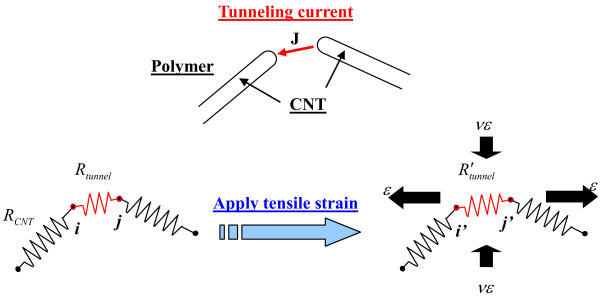
Modeling of tunneling effect among neighboring CNTs.

Moreover, considering the neighboring CNTs’ tunneling effect, we modeled CNTs as ‘hard-core’ objects instead of the so-called ‘soft-core’ model of fiber [15] to deal with both tensile and compressive strains on nanocomposites, with the assumption of non-penetration between the CNTs. As shown in Figure 3, for randomly distributed CNTs in a 3D unit cell, when the distance between two adjacent CNTs is smaller than the cutoff distance of 1.0 nm (e.g., zero, which means they are in contact with each other), the tunneling effect (resistance) is introduced. For example, at point *i* (Figure 3), if two CNTs are in contact or penetrate each other, we *intentionally* separate them with the shortest distance of 0.47 nm to avoid further penetration and then add tunneling resistance *R*_tunnel_ between them. This operation is very time-consuming for high CNT loading cases since there may be multiple contact points for one CNT with other neighboring CNTs. However, for low CNT loading cases discussed here, this operation is comparatively simple. The shortest distance (0.47 nm) is determined by matching the numerical predicted conductivity and piezoresistivity with the experimental ones. It is reasonable to set up this shortest distance (0.47 nm) since, in general, the equilibrium distance between two independent carbon atomistic structures under Lennard-Jones potential or van der Waals force ranges approximately from 0.3 to 0.5 nm. Furthermore, it is interesting to find that the shortest distance is much lower than the average diameter of the CNTs in the numerical simulations (i.e., 50 nm). Therefore, the range of distance *d* between two central lines of two cylindrical CNTs to introduce the tunneling effect should be *D* + *d*_min_*D* + *d*_max_, where *d*_min_ and *d*_max_ are 0.47 nm and 1.0 nm, respectively.

The resistance change caused by the tunneling effect due to an applied strain is schematically shown in Figure 3. The tunneling resistance between two neighboring CNTs can be approximately estimated as follows [17]:

(2) $$R_{\mathrm{tunnel}}=\frac{V}{AJ}=\frac{h^{2}d}{Ae^{2}\sqrt{2m\lambda }}\exp\left(\frac{4\pi d}{h}\sqrt{2m\lambda }\right)$$

where *J* is tunneling current density (see Figure 3), *V* is the electrical potential difference, *e* is the quantum of electricity, *m* is the mass of electron, *h* is Planck’s constant, *d* is the distance between CNTs, *λ* is the height of the barrier (for epoxy, 0.5 to 2.5 eV), and *A* is the cross-sectional area of the tunnel (the cross-sectional area of CNT is approximately used here).

Based on the well-known matrix representation for a resistor network [15] and Kirchhoff’s current law, the total electrical current *I* under an applied voltage can be estimated (see Figure 2). This is a large-scale linear system because the number of CNTs involved in the numerical model is very large, which ranges from several thousands, possibly up to several tens of thousands, depending on the aspect ratio of the CNTs. An iterative equation solver (i.e., the incomplete Cholesky conjugate gradient method) has been used to solve these linear equations for obtaining the total electrical current *I*. Then, the macroscopic electrical conductivity of the nanocomposites can be evaluated by Ohm’s law, which is used for predicting the resistance of the nanocomposites.

The reorientation of CNTs under the applied strain is simplified as a rigid-body movement due to a much higher Young’s modulus of CNTs compared with a polymer matrix and the very weak interface between CNTs and the matrix [6]. Therefore, the change of CNT position and orientation caused by the mechanical strain can be evaluated using the 3D fiber reorientation model [16] based on an affine transformation (see Figure 3). Corresponding to an updated CNT distribution under a prescribed mechanical strain, a new CNT network can be formed by re-calculating the possible intersections between CNTs and tunneling resistances between CNTs within the cutoff distance.

The switch of the CNT intersections to a possible tunneling effect due to the breakup of CNT contacts and the update distance of pre-existing tunneling effects were modeled. Then, the resistance of a nanocomposite at a fixed strain level can be re-evaluated using the 3D resistor network in an iterative way to finally calculate the piezoresistivity of the nanocomposite.

### Extension of the above model with considering the piezoresistivity of CNT

**Figure 4:**
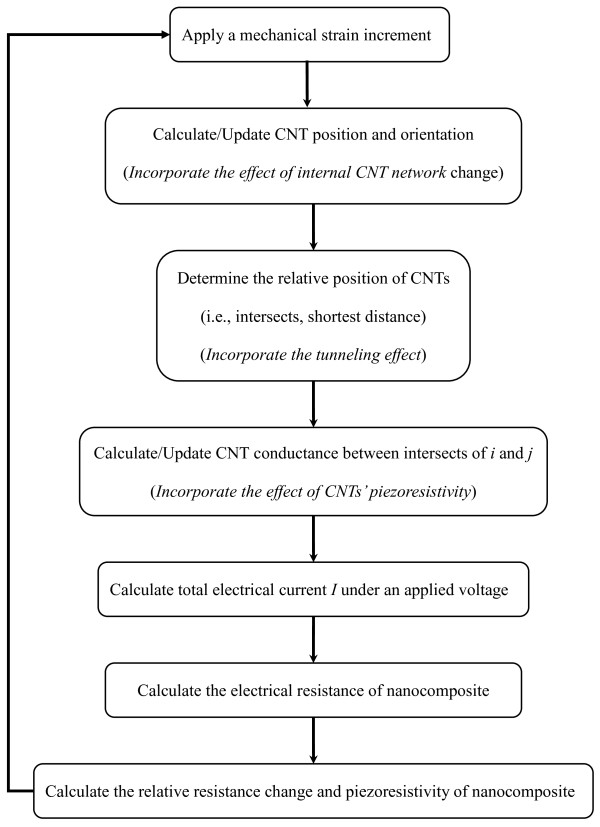
Flow chart of present extensive numerical model.

Here, the above model is extended to incorporate the effect of the piezoresistivity of CNTs themselves in a multi-scale way on the piezoresistivity of nanocomposite strain sensors, as well as the internal network change and the tunneling effect. The flow chart is summarized in Figure 4 with details as follows.

**Figure 5:**
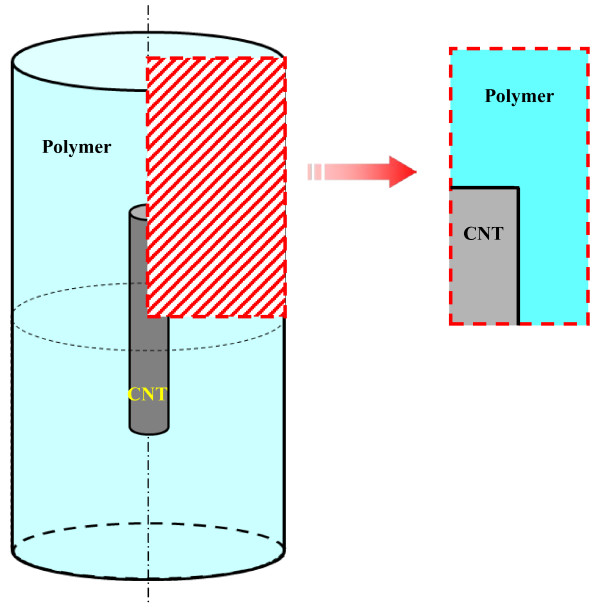
Axisymmetric RVE model.

**Figure 6:**
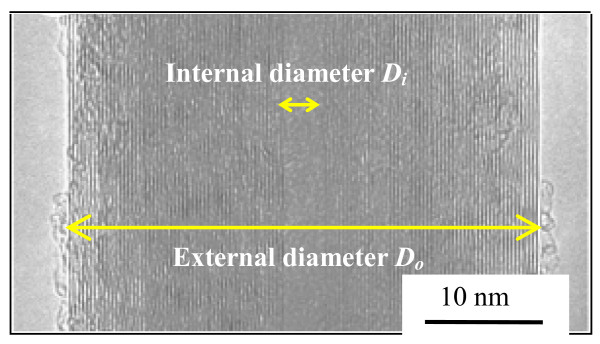
TEM image of a MWCNT.

**Figure 7:**
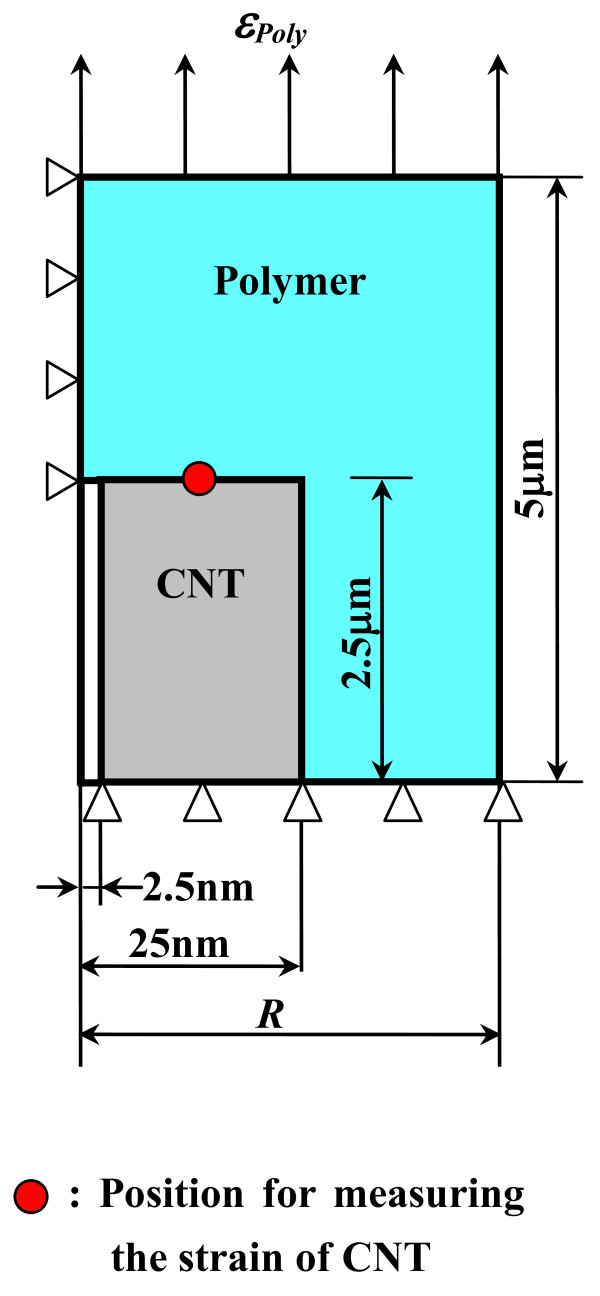
Dimension and boundary conditions of RVE model.

Firstly, the deformation characteristics of an arbitrary CNT embedded in a polymer nanocomposite should be clarified. As shown in Figure 5, for the case of one-directionally aligned CNTs, we pick up a CNT from nanocomposites and built a continuum-mechanics-based RVE consisting of the polymer matrix as an outer layer and an internally embedded CNT. To determine the size of this RVE, a MWCNT used in our previous experiments [6,7] was observed by a transmission electron microscope (TEM), as shown in Figure 6. The diameter of the MWCNT is approximately determined as 50 nm, and the ratio of the internal diameter *D*_i_ to the external diameter *D*_o_ is around 1:10. The length of the MWCNT is an average value provided by the maker (Nano Carbon Technologies Co., Ltd., Tokyo, Japan), i.e., 5 μm. The cap of the MWCNT was neglected in this RVE model. The sizes of the RVE are shown in Figure 7, where *R* is used as a parameter to adjust the CNT loading in nanocomposites. Moreover, the parameters used in the RVE are summarized in Table 1. The previously reported mechanical properties of CNTs and epoxy [6,7] were directly adopted.

**Table 1:** Parameters of the CNT and polymer

	**Parameter**	**Value**
CNT	Length (μm)	5
	Diameter (nm)	50
	Loadings (wt.%)	2, 3, 4, 5
	Young’s modulus (GPa)	1,000
	Poisson’s ratio	0.1
Polymer	Length (μm)	10
	Young’s modulus (GPa)	2.73
	Poisson’s ratio	0.34

**Figure 8:**
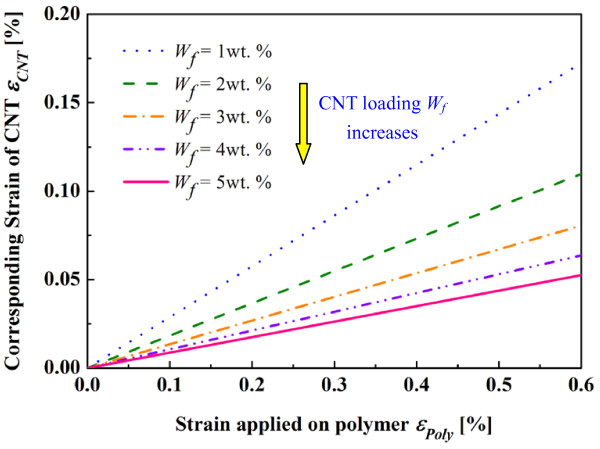
FEA results of the strain of CNT induced by the strain of polymer.

To perform the finite element analysis (FEA) on the RVE, as shown in Figure 7, the axisymmetric boundary conditions were applied on the RVE. The dimension and boundary conditions of the RVE is given in Figure 7 and Table 1. The case where the length of the polymer is two times longer than that of the CNT implies that the short carbon nanotubes are distributed evenly in both longitudinal and lateral directions in a matrix so that the RVE is the same for any CNT [18]. Moreover, under axial loading, some forces along the radial direction were imposed on the nodes of the outmost lateral surface of the RVE and adjusted through an iterative procedure so that all points on the outmost lateral surface move at the same distance in the radial direction to simulate the periodicity conditions [18]. When applying a strain *ε*_Poly_ ranging from 0.0 % to 0.6 % on the top surface of the polymer matrix, the strain of the CNT (i.e., *ε*_CNT_) at various CNT loadings can be calculated from the average axial displacements measured at the top surface of the CNT in the FEA. As plotted in Figure 8, the strain of the CNT is linearly proportional to that applied on the polymer, which can be expressed as:

(3) $$\varepsilon_{\mathrm{CNT}}=\alpha \varepsilon_{\mathrm{Poly}}$$

The strain ratio between CNT and polymer (i.e., *α = ε*_CNT/_*ε*_Poly_) at various CNT loadings are summarized in Table 2. It can be found that with the increase of CNT loading, *α* decreases significantly as the deformation of nanocomposites is transferred to and distributed on more CNTs.

**Table 2:** **Strain ratio*α*of CNT to polymer at various CNT loadings**

**CNT loading*W***_**f**_**(wt.%)**	**Strain ratio*α*in Equation3**
2	0.183
3	0.134
4	0.106
5	0.088

CNT, carbon nanotube; *W*_f_, CNT loading; *ε*_CNT_, strain of CNT; *ε*_Poly_, strain of polymer.

**Figure 9:**
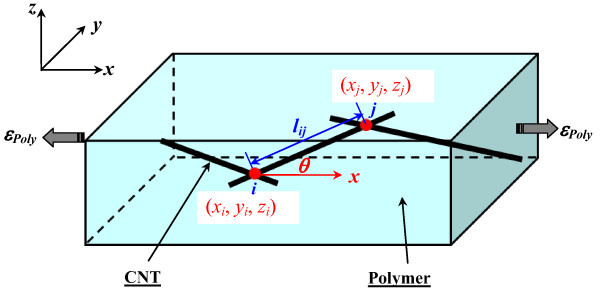
Schematic illustration of a randomly oriented CNT in polymer matrix.

Based on the above results, a randomly oriented CNT in macroscopic nanocomposites is then considered. Note that the relationship in Equation 3 corresponds to the simplest case of *θ* = 0 in Figure 9. For an oblique CNT with the strain of *ε*_*θ*_, we have

(4) $$\varepsilon_{\theta }=\varepsilon_{\mathrm{CNT}}\cos^{2}\theta$$

according to the coordinate transformation of strain by neglecting some other small strain components. Note that the above relationship is an approximate one since only the axial strain component was considered. To check its feasibility, another RVE was built in which an oblique CNT is embedded in a cylinder of epoxy, and the 3D FEA was also performed to obtain the axial strain of the oblique CNT induced by the axial strain of the RVE. It is found that Equation 4 can approximately evaluate *ε*_*θ*_ with only several percent errors by comparing with the FEA results stated above.

Provided that this arbitrary randomly oriented CNT located on a conductive path has two intersection points with two other neighboring CNTs, as shown in Figure 9, *θ* can be determined from the coordinates of the two intersection points as follows:

(5) $$\theta =\cos^{-1}\left(\frac{x_{j}-x_{i}}{l_{\mathrm{ij}}}\right)$$

where *l*_ij_ is the distance of the two intersection points calculated from (*x*_i_, *y*_i_, *z*_i_) and (*x*_j_, *y*_j_, *z*_j_).

After clarifying the deformation behavior of an arbitrary-oriented CNT in a nanocomposite under a mechanical strain, the corresponding resistance change and conductance of CNT can be obtained at the atomistic scale as follows: The resistance change of a SWCNT in some previous studies [13,14] seems to be not so obvious as obtained from the first-principle computations. For instance, the piezoresistivity at 0.6 % axial tensile strain is 3.84 % for a zigzag SWCNT(12,0) [13], 4.2 % for a SWCNT(8,1) [14], and −4.2 % for a SWCNT(8,0) [14]. For armchair SWCNTs, there is no piezoresistivity. For a MWCNT, we can approximately assume it as a SWCNT (i.e., the outermost wall of MWCNT) with a proper piezoresistivity. The contribution of the piezoresistivity of inner walls was neglected here due to their distance from the outermost wall, in which even the first inner wall is separated from the outmost wall by an interval of around 0.34 nm in an ideal state. Naturally, we do not know the quantity of the various types of outmost walls for the practically used MWCNTs in the experiments. By assuming that SWCNTs of various types are contained in equal quantities and all behave ideally, an average equivalent behavior of the SWCNTs is provided in [14], which indicates that the piezoresistivity of the SWCNTs is only around 1.27 % at 0.6 % axial tensile strain. This average equivalent behavior can be expressed as:

(6) $$\Delta \;R/R_{0}=2.118\varepsilon_{\theta }$$

where Δ*R* is the resistance change of a SWCNT due to a strain *ε*_*θ*_, and *R*_0_ is the initial resistance of this SWCNT.

The samples of SWCNTs used to obtain the average piezoresistivity response in Equation 6 were (8,0), (8,1), (8,2), (8,8), etc., and their diameters were very small [14]. For CNTs with large diameters, e.g., 50 nm in this work, the influence of diameter on Δ*R*/*R*_0_ can be neglected since Equation 6 only describes the resistance change caused by quantum effects due to CNT lattice deformation based on the Landauer formula, in which the size effect on Δ*R*/*R*_0_ is removed. Moreover, the influences of the changes of CNT’s length and cross-sectional area in classical electrical equations are neglected based on the following reasons: (1) they are very small under small strains, and (2) quantum phenomena are dominant at the atomistic level.

Furthermore, in Table 1, the Young’s modulus of the CNTs was taken as 1,000 GPa, which is slightly higher than most reported values for MWCNTs, e.g., 500 to 800 GPa. By using a lower Young’s modulus for a CNT, its strain should increase. However, by considering the weak piezoresistivity of CNTs in Equation 6 and low CNT loading, this strain increase cannot lead to remarkable piezoresistivity change of nanocomposite sensors.

The initial electrical conductivity of a CNT is expressed as follows:

(7) $$\sigma_{0}=\frac{1}{R_{0}}\frac{l}{S_{\mathrm{CNT}}}$$

where *S*_CNT_ is the cross-sectional area of a CNT, and *l* is the CNT length. Note that in this work, the initial electrical conductivity *σ*_0_ is taken as 1 × 10^4^ S/m.

When the CNT has a resistance change Δ*R* under a strain *ε*_*θ*_, its electrical conductivity can be described as:

(8) $$\sigma_{\mathrm{CNT}}=\frac{\sigma_{0}}{1+\Delta R/R_{0}}$$

By summarizing Equations 3 to 8, the electrical conductance in Equation 1 for the CNT segment between points i and j in Figure 7 can be rewritten into the following form to include the effect of the CNT’s piezoresistivity:

(9) $$g_{\mathrm{ij}}=\frac{\sigma_{0}}{1+2.118\alpha \varepsilon_{\mathrm{Poly}}\cos^{2}\theta }\frac{S_{\mathrm{CNT}}}{l_{\mathrm{ij}}}$$

On the other hand, when a strain *ε*_Poly_ is applied on a nanocomposite, the coordinates of the two intersection points i and j in the arbitrary CNT segment as shown in Figure 9 can be updated as follows by using the 3D fiber reorientation model [16] based on an affine transformation:

(10) $${x^{\prime }}_{i}=x_{i}\left(1+\varepsilon_{\mathrm{Poly}}\right)$$

(11) $${y^{\prime }}_{i}=y_{i}\left(1-\nu \varepsilon_{\mathrm{Poly}}\right)$$

(12) $${z^{\prime }}_{i}=z_{i}\left(1-\nu \varepsilon_{\mathrm{Poly}}\right)$$

and

(13) $${x^{\prime }}_{j}=x_{j}\left(1+\varepsilon_{\mathrm{Poly}}\right)$$

(14) $${y^{\prime }}_{j}=y_{j}\left(1-\nu \varepsilon_{\mathrm{Poly}}\right)$$

(15) $${z^{\prime }}_{j}=z_{j}\left(1-\nu \varepsilon_{\mathrm{Poly}}\right)$$

where *ν* is the Poisson’s ratio of the nanocomposite. Note that although we consider the resistance change of a strained CNT here, the changes of geometries of the CNT (e.g., cross-sectional area of CNT, i.e., *S*_CNT_, and CNT length, i.e., *l*) are neglected for simplicity in Equation 9 since basically, after the axial deformation with a strain *ε*, the changes of the length and cross-sectional area of a CNT can be described as *l* + *εl*, and *S*_CNT_-ν^2^*ε*^2^*S*_CNT_, which are very small for the maximum strain 0.6 % considered in this work. Therefore, in the above relationship for updating the coordinates, we also neglect the coordinate changes caused by the strain of the CNT. After obtaining the new coordinates of the intersections, we can calculate the new distance between i and j as:

(16) $${l^{\prime }}_{\mathrm{ij}}=\sqrt{{\left({x^{\prime }}_{j}-{x^{\prime }}_{i}\right)}^{2}+{\left({y^{\prime }}_{j}-{y^{\prime }}_{i}\right)}^{2}+{\left({z^{\prime }}_{j}-{z^{\prime }}_{i}\right)}^{2}}$$

By substituting Equations 10 and 11 into Equations 5 and 9, we can update the orientation angle of the CNT and the electrical conductance *g*_ij_ between i and j.

Note that besides update of the electrical conductivity of the CNT segment on the internal conductive network, we also update the internal CNT network by judging the loss or formation of contacting points, and update the tunneling effect by changing the distance among those neighboring CNTs, as done in our previous work [12].

## Results and discussion

The parameters for numerical simulations are defined in Table 3. Barrier height *λ* in Equation 2 was approximately determined by matching the zero-strain numerical electrical conductivities with the experimental ones at a reasonable range. To reduce the computational cost, the dimension of the unit cell (length × width × thickness) was set as 25 × 25 × 15 μm, which has been estimated to be large enough for an isotropic behavior and numerical convergence. The geometrical parameters of practical MWCNTs in our previous experiments [6,7] were directly adopted with length of *l* = 5 μm and diameter of *D* = 50 nm. For those CNTs with a mutual distance between 0.47 and 1 nm, the tunneling effect was incorporated between them, as stated previously.

**Table 3:** Computational conditions for present numerical simulation

	**Parameter**	**Value**
CNT	Length (μm)	5
	Diameter (nm)	50
	Electrical conductivity (S/m)	1 × 10^4^
	CNT loading *W*_f_ (wt.%)	2, 3, 4, 5
Barrier height *λ* (eV)		1.5
Tunneling distance range (nm)	Minimum value	0.47
	Maximum value	1
Unit cell size (μm^3^)		25 × 25 × 15

CNT, carbon nanotube; *W*_f_, CNT loading.

**Figure 10:**
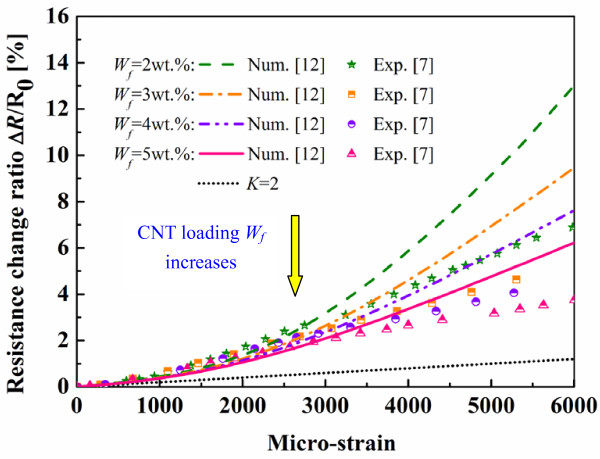
**Comparison of previous numerical results**[12]**and experimental ones**[7]**.** (Validation of previous numerical model [12]).

The average resistance change ratio predicted from 50 Monte Carlo simulations using our previous model [12] without considering the piezoresistivity of CNTs are shown in Figure 10 by comparing with our previous experimental results [7]. The resistance change ratio of traditional metal-foil strain gauges was also plotted (*K* = 2). In this figure, it can be seen that the numerical simulations can qualitatively catch the main trend of the experimental results under tensile strains although the numerical results are higher. The nonlinear behaviors of numerical and experimental piezoresistivities can be observed clearly. It implies that the tunneling effect plays a very important role in determining the overall performance of the nanocomposites at low CNT loading. According to Equation 2, a 0.1nm increase of the distance between two CNTs (i.e., *d*) can lead to ten times lower tunneling current (*λ* = 0.5 eV, *A* = π(*D*/2)^2^, and *D* = 50 nm). From Equation 2, the tunneling resistance increases exponentially with the average distance *d*. As the average distance *d* is approximately assumed to change proportionally with an applied strain, the nonlinear relationship between the resistance change ratio and the applied strain is expected. Especially for the cases of low CNT loadings and high strains, this nonlinear behavior becomes more obvious, which indicates the significance of tunneling effect. Moreover, as similar to some experimental studies [6,7,9], low CNT loading cases show large resistance change ratios, which indicates the increase of the sensor sensitivity. The reason can be explained as follows. Generally, the resistance change is mainly caused by the breakup of CNT conductive network or tunneling effects. For a sparse conductive network, the breakup of a few conductive paths can lead to a huge increase of sensor resistance, which results in the higher sensor sensitivity. Inversely, for an intensive CNT conductive network, the breakup of a few conductive paths cannot cause a significant change of total sensor resistance.

**Figure 11:**
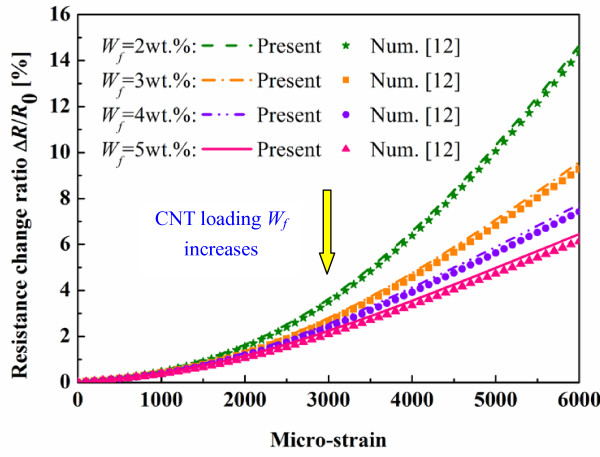
**Comparison of present numerical results with previous numerical results**[12]**.** (Effect of CNTs’ piezoresistivity on the total piezoresistivity of nanocomposite sensor).

On the other hand, the resistance change ratio predicted using the present numerical model with consideration of the CNTs’ piezoresistivity is shown in Figure 11 by comparing with those predicted by previous numerical model [12] in Figure 9. It was found that the macroscopic resistance change ratio of the nanocomposite sensors increases. However, the effect of the CNTs’ piezoresistivity on the total piezoresistivity characteristic of nanocomposite sensors is very limited as expected. At the maximum strain, i.e., 6,000 με, the piezoresistivity of CNTs only causes the maximum increase in the resistance change ratio of the sensors as 4.85 % (5 wt.%), 4.07 % (4 wt.%), 3.3 % (3 wt.%) and 2.11 % (2 wt.%), respectively, when compared with those neglecting the CNTs’ piezoresistivity. Moreover, it can be found that as the CNT loading increases, the effect of the CNTs’ piezoresistivity becomes more obvious, which can be attributed to the increasing number of CNTs in the nanocomposite sensors.

To further confirm the effect of CNTs’ piezoresistivity, by considering the possible variation in the previous data [14], we artificially amplify the piezoresistivity of CNTs in Equation 6 by ten times as follows:

(17) $$\Delta \;R/R_{0}=21.18\varepsilon_{\theta }$$

**Figure 12:**
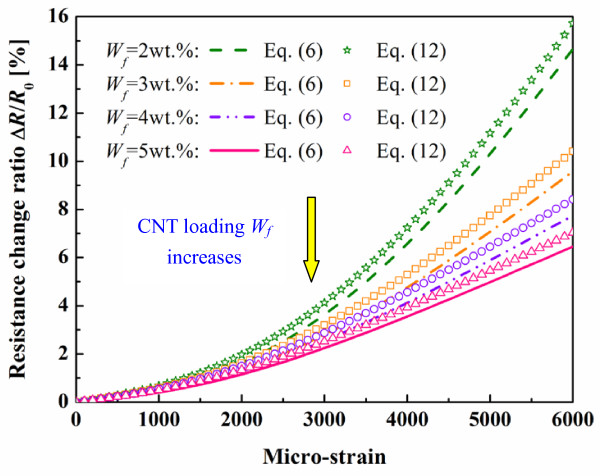
**Comparison of present numerical results using Equations6and12.** (Amplification of the effect of CNTs’ piezoresistivity).

This extreme case might be considered as the outmost walls of all MWCNTs are of the highest piezoresistivity. By using this new piezoresistivity of CNTs, we re-calculate the resistance changes of nanocomposite sensors and compare them with those obtained by Equation 6 in Figure 12. The effect of CNTs’ piezoresistivity on the total piezoresistivity of the nanocomposite sensors becomes more obvious. However, the maximum increase of the resistance change ratio at 6,000 με are only 8.88 % (5 wt.%), 8.8 % (4 wt.%), 8.65 % (3 wt.%), and 7.22 % (2 wt.%), respectively, when compared with those using Equation 6. Therefore, it can be concluded that the influence of the CNTs’ piezoresistivity on the total piezoresistivity of the nanocomposite sensors is limited when compared with the two other working mechanisms.

Note that the CNTs in the present numerical model are distributed uniformly at random without considering their possible aggregations. From our recent experimental results (NH et al., unpublished data), some moderate aggregations can lead to a sparse conductive network of CNTs, and then result in a higher sensor sensitivity. However, in this case, the stability and repeatability of nanocomposite sensors decrease especially for the case of low CNT loading. Moreover, we deal with the polymer matrix as a completely insulating one. Some polymers may be electrically conductive to a certain extent. In this case, this point may also influence the piezoresistivity of nanocomposite sensors. Although it is quite difficult to precisely estimate this influence, it can be speculated from the following two aspects. Firstly, by considering Ohm’s law, the resistance *R*_p_ of a polymer matrix only is proportional to *L*/*A*, where *L* and *A* are the length and cross-sectional area of a nanocomposite sensor. Then, *R*_p_ will change by Δ*R*_p_ since both *L* and *A* vary when the sensor is subjected to a strain. Therefore, the total piezoresistivity of the nanocomposite sensor should increase when considering the contribution of Δ*R*_p_. Secondly, the working mechanism, i.e., the change of internal network of the CNTs due to strain, might be weakened since the electric current can still pass through the matrix at the breakup points of the CNT network. However, when the electrical conductivity of the conductive polymer is much lower than that of the CNTs, this influence might be small since electric current always smartly chooses the components of much higher conductivity, i.e., CNTs, to pass through.

## Conclusions

In this work, we extend our previous 3D resistor network numerical model for predicting the piezoresistivity behaviors of strain sensors made from CNT/polymer nanocomposites. Besides the change of the internal conductive network formed by the conductive CNTs and the tunneling effect among neighboring CNTs, the contribution of the CNTs’ piezoresistivity was incorporated simultaneously. This modeling was realized by using a multi-scale technique in which we firstly compute the strain of a CNT in a RVE, then transform it into the strain of a randomly oriented CNT in the polymer matrix under a prescribed strain, and finally build up the relationship between the electrical conductivity change of the CNT using the above strain information and the CNTs’ piezoresistivity obtained from the first-principle computations [14]. An iterative model, which combines the 3D resistor network model and the fiber reorientation model, was constructed to predict the total piezoresistivity of the nanocomposite sensors from the updated information including CNT position, the internal conductive network, the tunneling effect among those neighboring CNTs, and the electrical conductivity of CNTs. The computational results qualitatively agree with our previous experimental results very well. Moreover, the contribution of the piezoresistivity of CNTs on the total piezoresistivity of the nanocomposite sensors is comparatively small, compared with those from the change of the internal conductive network and tunneling effects. The present numerical results can provide some valuable information for designing the highly sensitive resistance-type strain sensors made from the nanocomposites containing CNTs or other nanofillers.

## Competing interests

The authors declare that they have no competing interests.

## Authors’ contributions

BH and KA performed the numerical simulations. NH and YL designed the concept, analyzed the results and drafted, revised and finalized the manuscript with partial contribution from WFY, TW and YC. All the authors approved the final manuscript.
